# Recent advances in the electrochemical synthesis of organophosphorus compounds

**DOI:** 10.3762/bjoc.21.61

**Published:** 2025-04-16

**Authors:** Babak Kaboudin, Milad Behroozi, Sepideh Sadighi, Fatemeh Asgharzadeh

**Affiliations:** 1 Department of Chemistry, Institute for Advanced Studies in Basic Sciences (IASBS), Gava Zang, Zanjan 45137-66731, Iran

**Keywords:** electrosynthesis, green synthesis, organophosphorus compounds, P–C bond formation, P–heteroatom bond formation

## Abstract

In this review, we describe recent advances in electrochemical green methods for the synthesis of various organophosphorus compounds through the formation of phosphorus–carbon, phosphorus–nitrogen, phosphorus–oxygen, phosphorus–sulfur, and phosphorus–selenium bonds. The impact of different electrodes is also discussed in this matter. Graphite, platinum, RVC, and nickel electrodes have been used extensively for the electrochemical synthesis of organophosphorus compounds. The recent advances in the electrochemical synthesis of organophosphorus compounds have made this method a promising method for preparing various structures. This review is an introduction to encourage scientists to use electrosynthesis as a green, precise, and low-cost method to prepare phosphorous structures.

## Introduction

The electrochemical synthesis is a valuable and beneficial method for the preparation of organic compounds. In recent years, many advances have been made in this field of research, and researchers have been able to synthesize many compounds and confirm various uses for chemical compounds in this field. The significant progress in this field of research led to the name of this field as “greener chemistry”. Today, electrochemical synthesis has many applications in industry, and thousands of tons of chemicals are produced by this method every year [1–11].

In electrochemical synthesis, only electricity is used instead of oxidizing or reducing substances. Electricity can perform the oxidation and reduction process by exchanging electrons on the electrode surface in a region called the double layer (DL) [12]. Unlike traditional methods that require high temperature, pressure, and external oxidants, electrochemistry is an efficient and energy-saving approach that controls reaction selectivity by adjusting voltage or current [13]. Simple synthetic systems in electrochemical methods are limited to electrodes, cells, electrolytes, and power supplies. Today, in addition to the above, light, metallic, and organic catalysts are also used to increase the efficiency of reactions [14–21].

Organophosphorous compounds are essential materials with broad applications in medicinal chemistry, synthesis, agriculture, as ligands, and intermediates to prepare complex compounds. Due to their importance, scientists have introduced many studies in recent years on developing new methods for synthesizing organophosphorus compounds [22–35].

Developing sustainable and green methods for synthesizing organophosphorus materials is a growing field. Methods based on photocatalysis [36], flow-based technologies [37–38], and microwave irradiation [39–42] have been developed. The electrochemical synthetic method is a creative, simple, and new process for preparing organophosphorus compounds [43].

In recent years, various articles have been reported on the electrochemical synthesis of organophosphorus compounds, in which phosphorus is attached to carbon or heteroatom centers. In this article, we describe recent advances in electrochemical green methods for the synthesis of various organophosphorus compounds through the formation of phosphorus–carbon, phosphorus–nitrogen, phosphorus–oxygen, phosphorus–sulfur, and phosphorus–selenium bonds. The impact of different electrodes is also discussed in this matter. Graphite, platinum, reticulated vitreous carbon (RVC), and nickel electrodes have been used extensively for the electrochemical synthesis of organophosphorus compounds.

## Review

### Electrochemical reaction cells

When a redox reaction occurs indirectly, chemical energy is transformed into electrical energy. A device that facilitates this conversion is known as an electrochemical cell. Electrochemical reaction cells are divided into two primary categories: galvanic (voltaic) and electrolytic. They consist of two electrodes – anode (where oxidation occurs) and cathode (where reduction occurs) – immersed in an electrolyte.

#### Galvanic cell

The redox reaction occurs spontaneously in these cells, converting chemical energy into electrical energy. The potential difference between the two electrodes generates an electric current. Some of its applications include batteries (e.g., lithium-ion batteries) and fuel cells. These cells are usually in a divided state.

#### Electrolytic cell

These cells require an external voltage to drive chemical reactions. They use electrical energy to carry out a non-spontaneous reaction. Some of their applications include hydrogen and oxygen production, metal electroplating, and organic compound synthesis using electrochemical methods. Depending on the reaction conditions, these cells can be divided or undivided.

#### Divided vs undivided cells

In divided cells, oxidation and reduction occur in separate compartments, separated by a diaphragm or salt bridge, to prevent reactant mixing and improve efficiency (e.g., Daniel cell). However, in undivided cells, both reactions occur in a single compartment without separation, resulting in a more straightforward design but potentially lower efficiency (e.g., some electrolytic cells).

#### Role of electrolytes in organic electrochemical reactions

Electrolytes are crucial for conductivity and reaction stability in organic electrochemical reactions. They are categorized as supporting electrolytes, which enhance conductivity, reduce resistance, and maintain ion balance (e.g., LiClO_4_, *n*-Bu_4_NBF_4_), and active electrolytes, which participate directly in redox reactions, acting as oxidizing or reducing agents (e.g., H_2_SO_4_, Et_4_NOH). Choosing the proper electrolyte affects reaction efficiency, selectivity, and overall performance.

#### Electrodes in the synthesis of organophosphorus compounds

Electrodes (any conductive materials) are one of the vital components in electrochemical cells (divided or undivided cells) for synthesizing organic compounds. The results of any electrosynthesis process depend entirely on the contact surface of the electrode with the reaction solution. The oxidation–reduction process complements each other, and the surface of the electrode in the reaction is critical. The material of the electrode is essential [44]. Various electrodes, including carbon (C), platinum (Pt), nickel (Ni), and reticulated vitreous carbon (RVC), are extensively used in the electrosynthesis of organophosphorus compounds (Table 1).

**Table 1:** Electrodes used in the electrosynthesis of organo-phosphorus compounds.

Material	Anode	Cathode

C	62%	12%
Pt	31%	74%
Ni	–	14%
RVC	5%	–

**Carbon (C) electrode:** The carbon electrode is one of the most widely used electrodes in electrochemical synthesis. This electrode is a porous material that allows chemicals to penetrate it. On the other hand, this electrode is one of the inexpensive electrodes. Usually, the carbon electrode needs to modify the surface, which allows for more straightforward chemical modification by installing it on other electrodes. The high fragility of the carbon electrodes and the difficulty of their cleaning are disadvantages of these electrodes. A wide range of electrodes, such as graphite, glassy carbon, and pyrolytic carbon, are based on carbon. In synthesizing organophosphorus compounds by electrochemical methods, more than 60% of the anodes were made of carbon. The carbon electrode did not respond well as a cathode and was used only in ≈10% of the synthesis of organophosphorus compounds by electrochemical methods.

**Platinum (Pt) electrode:** The platinum electrode has an extensive oxidation range, is difficult to enter into the reaction, and can be very inert. This electrode is beneficial in electrosynthesis processes and can work well as the anode and cathode. This electrode has high stability in the electrochemical environment and is easy to clean, but caution should be taken when using it as a cathode because of low H_2_ overpotential. Platinum electrodes are very popular and valuable as cathodes in the electrochemical synthesis of organophosphorus compounds. They are used as the cathode in more than 70% and as the anode in ≈30% of electrosynthesis processes.

**Nickel (Ni) electrode:** Nickel is not usually used as the anode but as a sacrificial anode in electrosynthesis. Using nickel as the cathode has a better performance, and it has not been used as the anode in the electrosynthesis processes of organophosphorus compounds.

### Electrochemical synthesis of organophosphorus compounds

#### Electrochemical C–P bond formation

**Scheme 1:**
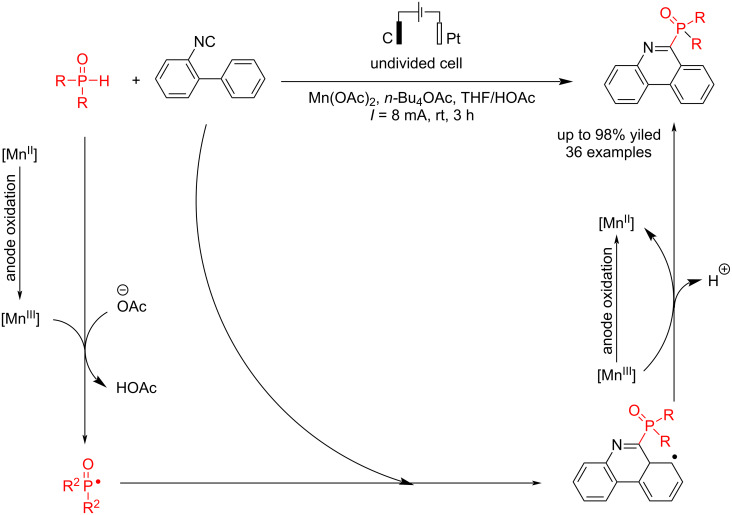
Electrosynthesis of phenanthridine phosphine oxides.

Various articles on the electrochemical synthesis of organophosphorus compounds have been reported in recent years. Recently, an electrochemical reaction of 2-isocyanobiaryls with diphenylphosphine oxides has been reported by Li et al. [45] using a Mn catalytic system with C(anode)/Pt(cathode) in an undivided cell. Different products were obtained with up to 85% yield in a constant flow for three hours. Studies showed that a Mn catalyst is critical for synthesizing derivatives of phenanthridine-based diarylphosphine oxides. The reaction yield decreased in the absence of the ligand, and eliminating both the ligand and manganese salt suppressed the reaction. Moreover, a slight decrease in the reaction yield was observed with increased reaction temperature. 2-Isocyanobiaryl compounds showed better reactivity when they contained electron-withdrawing groups. Diarylphosphine oxides containing a methyl group reacted well under standard conditions, regardless of their position. Mechanistic studies showed that when the reaction was carried out in the presence of TEMPO as a radical scavenger, a side product, TEMPO-P(O)R_2_, was formed (it was confirmed using high-resolution mass spectrometry). The results revealed that the reaction proceeded in a radical pathway (Scheme 1). Based on the cyclic voltammetry experiments, the oxidation current increased further with the addition of diphenylphosphine oxide to the mixture of Mn(OAc)_2_ and 2,2-bipyridine. This suggests that 2,2-bipyridine likely influenced the oxidation of Mn(II) and reacted with diphenylphosphine oxide. Further studies have also shown that the platinum electrode as a cathode is very suitable for the process. Increasing the contact surface of the anode using a graphite felt electrode instead of a graphite rod in the reaction medium was one of the ways to improve the result.

**Scheme 2:**
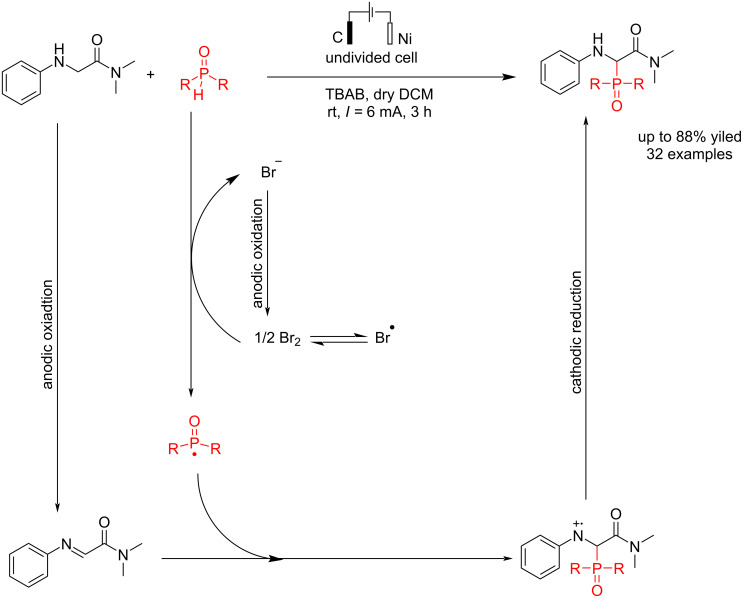
Electrosynthesis of 1-aminoalkylphosphine oxides.

In 2023, Wang et al. [46] reported an electrochemical reaction of amide derivatives of glycine with diarylphosphine oxide (R_2_P(O)–H) for the synthesis of 1-aminoalkylphosphine oxides without the use of any transition metal catalyst or external oxidant. In this conversion, 1-aminoalkylphosphine oxides were formed in an undivided cell using a carbon electrode as the anode and nickel as the cathode in the presence of tetrabutylammonium bromide (TBAB) at the constant current of 6 mA. The electrodes used in the reaction are all in plate form. The presence of TBAB causes the resulting bromide anion to oxidize to bromine radical and react with R_2_P(O)–H to produce a radical phosphorus center. The reaction yield was higher when nickel was used as the cathode and graphite as the anode compared to the setup where nickel was replaced with graphite for the cathode and/or graphite was replaced with nickel for the anode (Scheme 2). This method is suitable for phosphorylating glycine amides with electron-withdrawing or electron-donating groups on their aromatic ring, producing products with yields ranging from 51% to 82%.

**Scheme 3:**
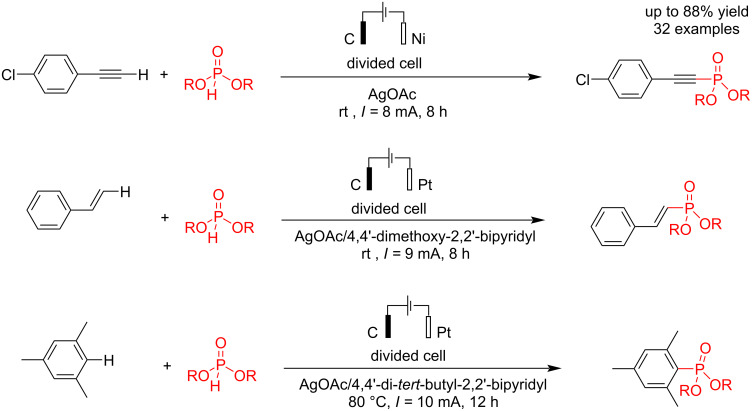
Various electrochemical C–P coupling reactions.

In 2023, Lei and co-workers [47] reported an electrochemical C–P bond formation via a coupling reaction of C–H bonds of alkynes, alkenes, and aryl compounds with dialkyl phosphonates at carbon and platinum electrodes as the anode and cathode in the presence of a silver catalyst in a divided cell (Scheme 3). According to the report, the silver catalyst is central to the coupling reaction. The study of the effect of alternating current (a.c.) electrolysis parameters on silver-catalyzed C–H phosphorylation revealed that variations in current intensity, frequency, and duty ratio influence product yield. To achieve optimal reactivity, the duty ratio must exceed 50%. Additionally, an analysis of silver deposition on carbon and platinum electrodes indicated that silver accumulation is minimal in alkynylation and arylation processes. In contrast, silver deposition was observed on the platinum electrode surface in alkenylation reactions. The article does not provide a mechanistic description of the reaction. Further investigations suggested that modifying the shape and contact area of the electrodes led to different reaction outcomes.

**Scheme 4:**
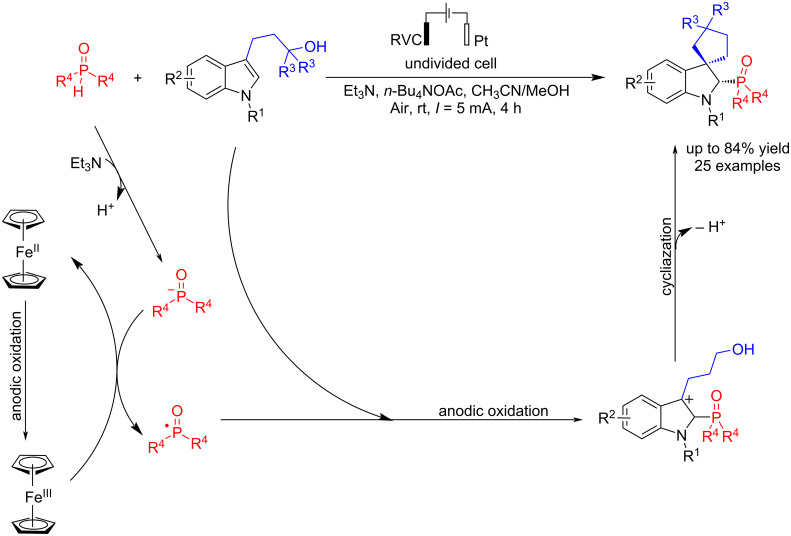
Electrochemical C–P coupling reaction of indolines.

Spirocyclic indolines have broad applications in medicine. The phosphorylated structures of indolines have also played a significant role in synthesis and industry. In 2023, Mo et al. [48] reported the electrochemical synthesis of a wide range of these compounds (25 compounds of phosphonylated 3,3-spiroindolines) in a convenient process. The C–P bond formation reaction of phosphine oxides with *N*-Boc-indolines was carried out in the presence of Cp_2_Fe, but the reaction did not occur in its absence. When the reaction was performed in the presence of TEMPO, the product of TEMPO-P(O)R_2_ was formed, which showed that the process proceeded through a radical path. In addition, the reaction was carried out in an electrochemical environment with an undivided cell, using graphite and platinum plate electrodes as the anode and cathode at constant current for 1 hour (Scheme 4). Platinum is usually used as the cathode for its ease of use, and a carbon plate electrode as the anode because of its cheapness and stability at high voltages. Further studies showed that using a reticulated vitreous carbon (RVC) electrode as the anode gave much better results than a graphite plate. The desired product was likely not formed due to the higher oxidation potential of diethyl phosphonate. Cyclic voltammetry experiments confirmed that Cp_2_Fe is oxidized first due to its lower oxidation potential than other compounds. Moreover, the reaction proceeded smoothly without Et_3_N or acetate, indicating that these compounds do not play a role in the reaction process (Table 2).

**Table 2:** Optimization studies.

Variation from the standard conditions	Yield (%)

none	62
without Cp_2_Fe	n.r.
without Et_3_N	31
CH_3_CN/HOAc instead of CH_3_CN/MeOH	22
CH_3_CN/H_2_O instead of CH_3_CN/MeOH	22
RVC(+)|Pt(−) instead of C(+)|Pt(−)	74
without electricity	n.r.

**Scheme 5:**
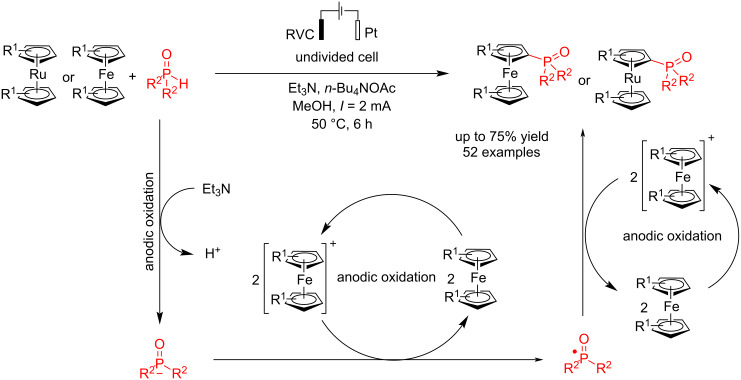
Electrochemical C–P coupling reaction of ferrocene.

To study ferrocene's electrochemical direct phosphorylation reaction with diphenylphosphine oxide, Chen et al. [49] examined and reported electrochemical C–P bond formation of ferrocene and ruthenocene via coupling reaction. This method provides an efficient and versatile synthetic approach for producing phosphorylated metallocenes but also aids in interpreting the regioselectivity and reactivity of C−H functionalization in unsymmetric metallocenes. They used a platinum electrode as the cathode and changed the anode electrode to find the best efficiency. The platinum plate electrode is more suitable than felt due to its larger contact surface, and using graphite electrodes in the form of a rod had good efficiency. The best results were obtained using a RVC electrode as the anode at a constant current at 50 °C in methanol for 6 h. This method also obtained a small amount of over-phosphorylated products. Phosphorylation occurred even when no functional group was present at the α-position of the aryl ring. Products with good yields were synthesized despite methyl, bromine, or phenyl groups at the α-position. The researchers noted that the lower yields were due to substrate decomposition or poor conversion rather than regioselectivity issues. Additionally, this method was also suitable for the phosphorylation of ferrocenes. For mechanistic studies, the reaction was examined in the presence of TEMPO as a radical scavenger, and the results revealed that the reaction proceeded through a radical pathway. The results of control experiments suggested that the phosphorylation might proceed through ferrocenium. It should also be noted that ferrocene and ruthenocene compounds have a low oxidation potential and can be oxidized quickly to act as a catalyst (Scheme 5).

**Scheme 6:**
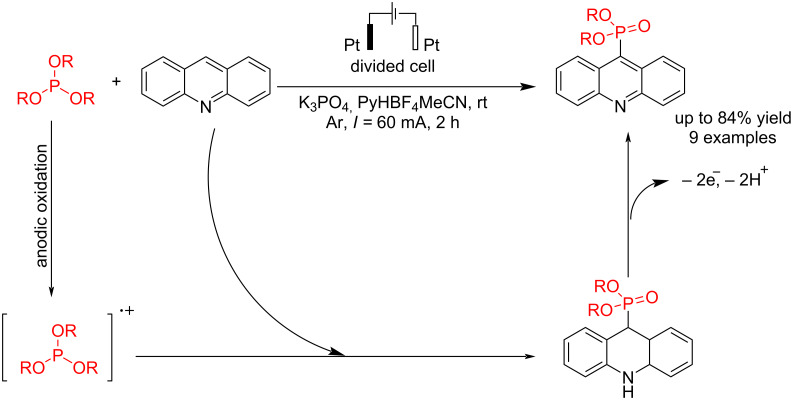
Electrochemical C–P coupling reaction of acridines with phosphites.

Acridines are important nitrogen-containing heterocyclic compounds used as the building block for preparing medicinally active compounds. The conjunction of phosphorus with acridine increases its biological activities. Budnikova et al. [50] reported a C–P bond formation via the reaction of acridine compounds with trialkyl phosphites in electrochemical conditions without metal catalysts and strong oxidizing reagents, conducting selective C9 phosphorylation with high yield. The reaction was carried out in an undivided cell at room temperature, and three different electrodes, graphite, platinum, and glassy carbon (GC), were examined during the reaction. The best result was obtained when platinum electrodes were used as the anode and cathode. Although the reaction was less efficient in undivided cells, increasing the electricity passed improved the reaction yield. The use of commercial acetonitrile without additional drying reduced the yield of the target product due to the formation of byproducts (RO)_3_PO and (RO)_2_P(O)H. It suggested that the reaction proceeded via anodic oxidation of trialkyl phosphite followed by treatment with acridine to give the corresponding coupling product (Scheme 6).

**Scheme 7:**
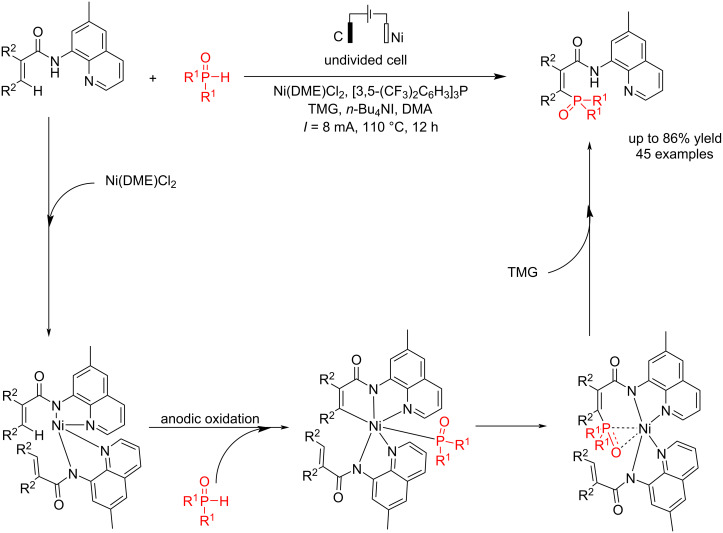
Electrochemical C–P coupling reaction of alkenes.

Vinylphosphonates have many applications in pharmaceutical, agricultural, and industrial processes. Zhang et al. [51] reported a novel electrochemical C–P coupling of specific alkenes with different types of phosphonates and phosphine oxides using a nickel catalyst. The use of nickel complex is an important and primary factor in the C–P coupling process. Notably, the inexpensive and environmentally readily available nickel catalysis was more effective for phosphorylation than other 3D metals. Moreover, it exhibited higher stability compared to 4d and 5d transition metals. Through the study of a series of previous experiments, it was shown that the electron density of the nitrogen atom in the quinoline structure significantly affects the efficiency of nickel-electrocatalysis; however, other *N*,*N*- or *N*,*O*-bidentate groups were unable to accelerate this reaction. To perform the reaction in an electrochemical environment, they used graphite (felt form) and nickel (nickel foam) electrodes as the anode and cathode, respectively, under a constant current of 8 mA at 110 °C. A non-radical reaction mechanism process was proposed by conducting the reaction in the presence of TEMPO (Scheme 7). The electron-deficient and sterically encumbered diaminophosphine oxide could also produce the corresponding products in this method. Gas chromatography analysis confirmed that molecular hydrogen was the only byproduct of this process.

**Scheme 8:**
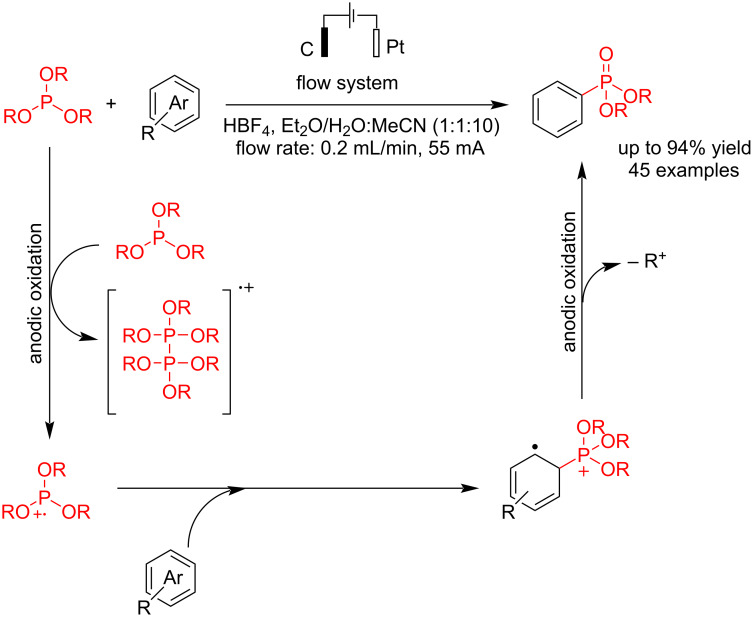
Electrochemical C–P coupling reaction of arenes in a flow system.

Arylphosphonates are essential compounds with a wide range of applications in pharmaceutical, biological, and agricultural materials. Therefore, finding new methods for preparing arylphosphonates is a significant challenge for scientists. Usually, metal catalysts are used to synthesize arylphosphonates via carbon–phosphorus bond formation. In 2021, Xu et al. [52] reported an electrochemical process for synthesizing arylphosphonates through the hetero-coupling reaction of CH of arenes with a trialkyl phosphite. They have prepared 45 arene phosphonates with good to excellent yields and reported the gram-scale preparation of some samples. An electrochemical flow system was used in this method, in which carbon and platinum electrodes were used as the anode and cathode, respectively, at a constant current of 55 mA (Scheme 8). Due to the steric hindrance caused by the *tert*-butyl group, the reaction occurred at the *ortho* position relative to the ester group. Also, after a few hours, the reaction yield decreased when the reactants were pre-mixed with HBF_4_. A series of analyses revealed that P(OEt)_3_ decomposes into various phosphorus species without H_2_O. Additionally, the studies showed that P(O)(OR)_2_ is derived from the compound P(OR)_3_, not from HP(O)(OR)_2_. Although the exact role of HP(O)(OR)_2_ remains unclear, it has been established that its presence is essential for the C–H phosphorylation. In this case, a radical cation intermediate was suggested for this conversion.

**Scheme 9:**
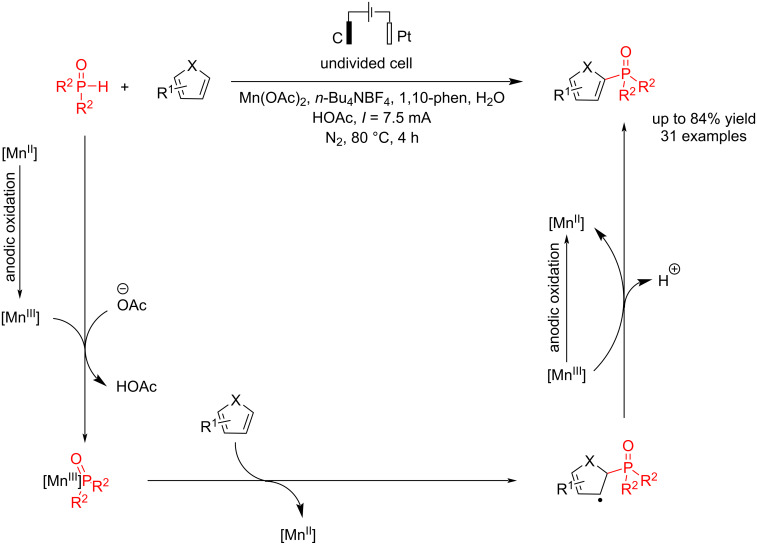
Electrochemical C–P coupling reaction of heteroarenes.

Heteroaromatic compounds such as furan and thiophene can be critical materials if attached to the phosphorus group. Wang et al. [53] reported an electrochemical process for the coupling of five-membered heteroaromatic rings with the P–H bond of diarylphosphine oxide in the presence of Mn(OAc)_2._ This report found that using manganese acetate as a catalyst is essential, and the reaction failed to produce any product without the catalyst. Other catalysts besides Mn were tested, but they showed poor reactivity. Other strong polar solvents were also used in this method, but they resulted in lower yields of the products. The reaction was carried out in an undivided cell with a graphite rod electrode as the anode and platinum as the cathode at a constant current of 7.5 mA under N_2_ for 4 h. Thiophenes with strong electron-withdrawing groups, and halogens produced moderate yields. However, a good yield was achieved when more thiophene and catalyst were added. On the other hand, heteroaromatics could not generate the corresponding products, likely due to their electron-rich nature and the presence of the active N–H group. The researchers noted that just one methyl group did not affect the reactivity. However, good-yield products were obtained when two methyl groups were positioned at the *para* or *meta* positions. The evaluation of the synthetic potential of the C−P bond formation revealed that the cleavage of the C−H bond in thiophene likely does not participate in the rate-determining step. Based on the experiments, a radical process was proposed for this coupling reaction via an Mn(III)–P intermediate (Scheme 9). The method was also applied to scale up to gram-scale synthesis.

**Scheme 10:**
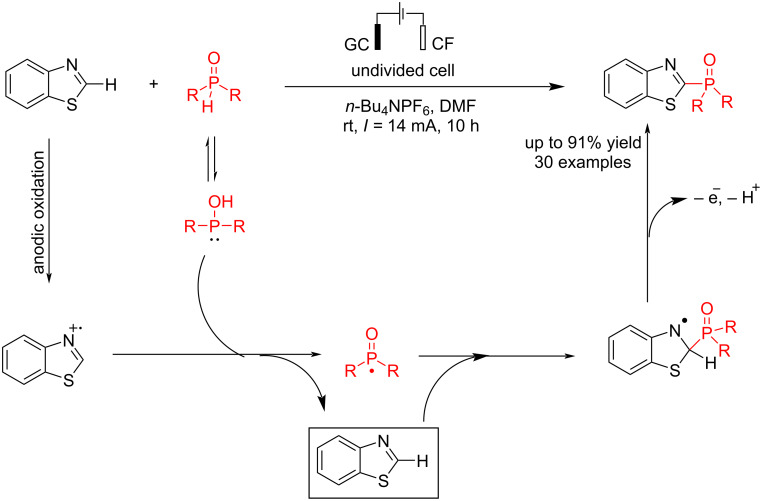
Electrochemical C–P coupling reaction of thiazoles.

In 2023, Wu et al. [54] also reported another heteroaromatic C–P coupling of benzothiazole with diarylphosphine oxides by an electrochemical method. They have synthesized 30 different thiazole phosphine oxides with up to 91% yield at room temperature without using an external metal or oxidant. The reaction was carried out in an undivided cell using glassy carbon as the anode and foamed copper as the cathode electrodes at a constant current of 14 mA for 10 h (Scheme 10). Other electrodes, including platinum, nickel foam, and graphite, were also examined for this reaction. The reaction failed to give the corresponding product using graphite as the anode and platinum as the cathode. The reaction showed lower efficiency under a nitrogen atmosphere, indicating that anodic oxidation is the main pathway of the reaction, and oxygen may have a positive effect. (Table 3). Functional groups at the 4-position moderately reduced the reaction yield. The nitro group was incompatible in this system, likely due to its preferential reduction ability. Some other heteroarenes were also tested, but only quinoxaline was compatible with this system under the standard conditions. A radical pathway was proposed in this reaction. At first, a thiazole radical cation was formed via anodic oxidation, followed by a reaction with phosphine oxides to give a phosphine oxide radical. The coupling product was obtained via the reaction of a phosphine oxide radical with thiazole compound.

**Table 3:** Optimization studies.

Variation from the standard conditions	Yield (%)

none	91
C (+), Pt (−)	16
Pt (+), Pt (−)	22
CH_3_OH instead of DMF	n.r.
CH_3_CN instead of DMF	10
under N_2_	84
without electricity	n.r.

**Scheme 11:**
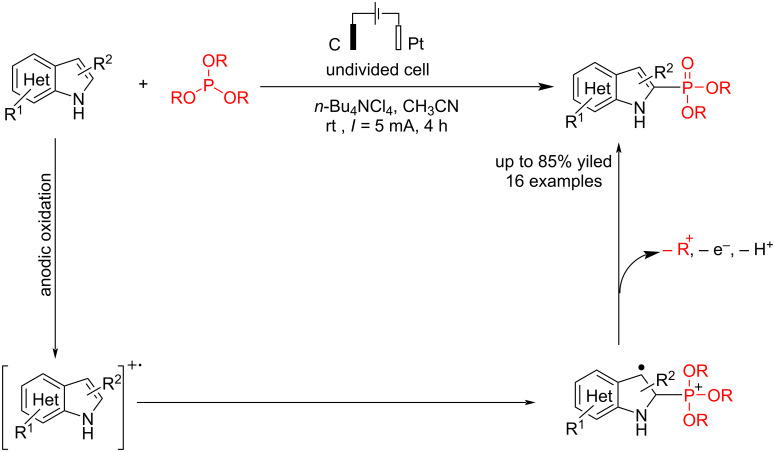
Electrochemical C–P coupling reaction of indole derivatives.

In another study on heteroaromatic compounds' electrochemical C–P coupling reactions, Gao et al. [55] reported an electrochemical reaction of indole derivatives with trialkyl phosphite in an undivided cell. The C–P product was selectively produced using *n*-Bu_4_NClO_4_ as electrolyte and carbon and platinum electrodes as the anode and cathode at a constant current for 4 h. Using *n*-Bu_4_NI instead of KI resulted in a similar outcome, but KBr was less effective (Table 4). The desired C2-phosphorylated indole was obtained with high selectivity when *n*-Bu_4_NClO_4_ was used as the electrolyte. Additionally, under certain conditions that reduced the reaction yield, the C3-phosphorylated product was also observed. Similar to previous heteroaromatic coupling reactions with phosphine oxides [53–54], this reaction proceeded via anodic indole oxidation, followed by a reaction with trialkyl phosphite to give the corresponding indole phosphonate (Scheme 11). Cyclic voltammetry experiments confirmed that free indole can oxidize at the anode and generate a radical-cation intermediate. Also, no product was detected when HP(O)(OEt)_2_ was used as the starting material.

**Table 4:** Reaction parameters.

Electrolyte	P(OE)_3_	Yield (%)

KI	3 equiv	n.d.
*n*-Bu_4_NI	3 equiv	n.d.
KBr	3 equiv	n.d.
*n*-Bu_4_NClO_4_	4 equiv	75
*n*-Bu_4_NBF_4_	4 equiv	30
NaBF_4_	4 equiv	58
KI	3 equiv	n.d.

**Scheme 12:**
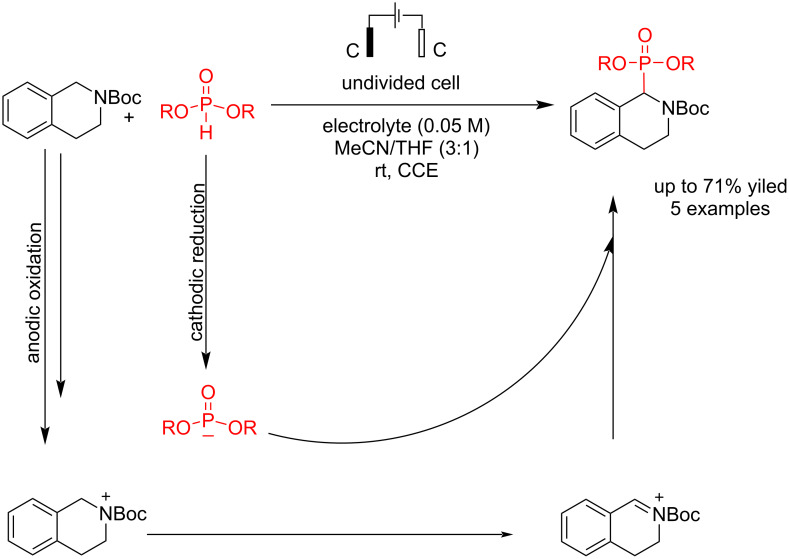
Electrosynthesis of 1-amino phosphonates.

Electrosynthesis processes of tetrahydroisoquinoline usually have a lower yield in the final product due to two electroactive positions in the molecule. Sengmany et al*.* [56] reported an electrochemical C–P bond formation of *N*-Boc-tetrahydroisoquinoline with dialkyl phosphites for synthesizing 1-amino phosphonates. The reaction was carried out at a constant current using graphite electrodes in both the anode and cathode. The optimal current intensity was observed when a 10 mA current was applied to the system. At higher current intensities, the reaction yield slightly decreased, and the formation of the Boc-deprotected product increased. When the reaction was performed in acetonitrile without THF, a greater quantity of the Boc-deprotected product was produced, which led to its degradation. This can be attributed to the Boc-deprotected compound being more easily oxidizable than the initial THIQ-N-Boc. Conversely, increasing the amount of THF relative to acetonitrile had the opposite effect on the yield. The use of diisopropyl phosphite decreased the reaction yield, which is presumed to be due to its steric effects. Moreover, phosphorylation did not occur with diphenyl phosphite, which can be attributed to its oxidizability. Unlike other phosphites, diphenyl phosphite is more easily oxidized than THIQ-*N*-Boc, preventing the coupling reaction. The reaction proceeded by coupling a cation intermediate of *N*-Boc-tetrahydroisoquinoline at the anode and phosphonate anion at the cathode (Scheme 12).

**Scheme 13:**
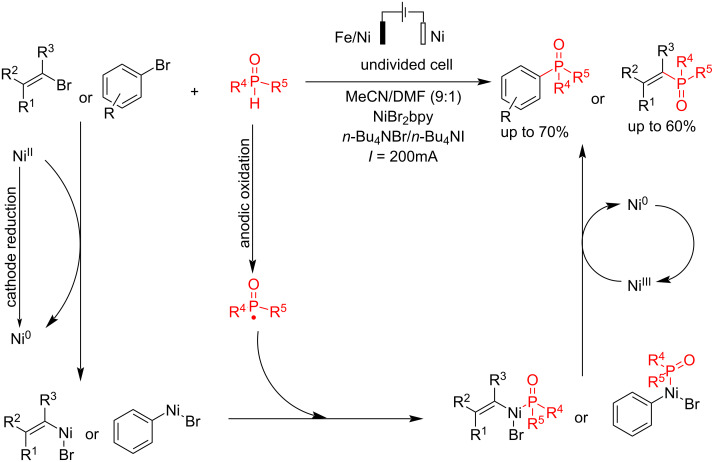
Electrochemical C–P coupling reaction of aryl and vinyl bromides.

The C(sp^2^)–X in aryl and vinyl halides is suitable in organic coupling reactions that are usually active in electrochemical environments. The use of combined electrodes is one of the creative methods in electrosynthesis processes. Léonel et al. [57] reported an electrochemical coupling reaction of aryl and vinyl bromides with different types of alkyl H-phenylphosphinates in the presence of NiBr_2_ as a catalyst. The reaction was carried out with an alloy of Ni-Fe as the anode and nickel as the cathode in an undivided cell at a constant current for 0.5–2 h (Scheme 13). It should be noted that the reaction failed to give good results using Ni or Fe as the anode (<10%). However, using an iron/nickel alloy electrode with 64% iron and 36% nickel gave good results. This method can also be applied to heteroaromatic bromides, although it shows an increased tendency for hydro-dehalogenation. Tetrabutylammonium halide was chosen as the electrolyte due to its lower hygroscopicity and reduced tendency for reductive homocoupling of 4-bromobenzotrifluoride. In the presence of chlorinated substituents, neither the double coupling product nor the hydro-dechlorination product was observed. This notable result suggests performing a second coupling using conventional chemical methods, such as the Suzuki–Miyaura reaction. Furthermore, the coupling yield decreased for phenyl bromides bearing bulky *ortho*-substituents while hydrodehalogenation byproducts formed. The reaction proceeded via an oxidative addition and reductive elimination processed in the presence of Ni(0), which was produced in situ from NiBr_2_ in the cathode.

**Scheme 14:**
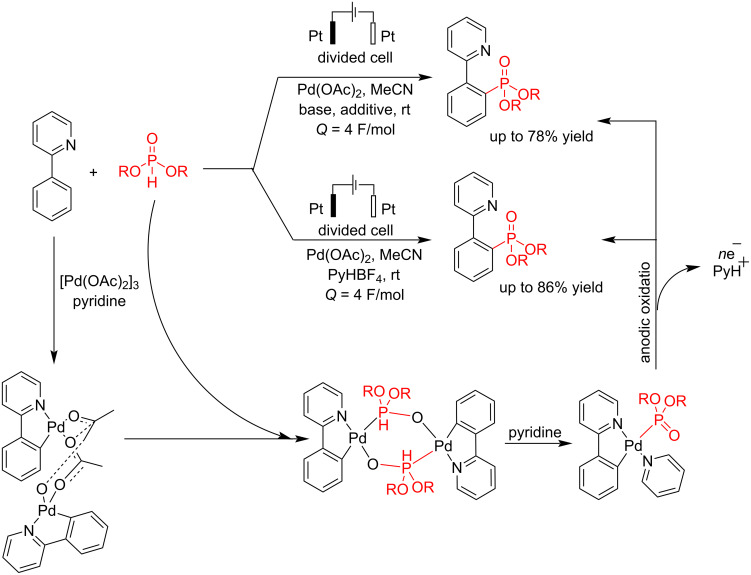
Electrochemical C–P coupling reaction of phenylpyridine with dialkyl phosphonates in the presence of Pd.

Palladium is one of the most important metals used as a catalyst in non-electrochemical reactions. In 2020, Budnikova et al. [58] reported a coupling reaction of phenylpyridine with dialkylphosphonate in the presence of palladium. It should be noted that the presence of palladium is an essential factor, and the results showed that the coupling reaction failed in the absence of palladium. Under pyridine-mediated conditions, the reaction follows a mononuclear palladacycle pathway, where a high electrolysis potential facilitates the formation of the *ortho*-phosphonate product with a favorable yield. On the other hand, when acid was used, forming a tetranuclear palladium intermediate led to the creation of a C−O−P bond. This reaction was carried out in a divided cell using platinum electrodes as the anode and cathode in the presence of pyridine as a base and ligand (Scheme 14). The catalyst behavior of palladium is attributed to its ability to form palladium clusters of specific sizes that exhibit high catalytic activity. However, this can lead to lower reaction yields because various reaction pathways, including those involving unstable metal-organic intermediates, may become involved. Cyclic voltammetry analysis in both solution and solid phases, using a carbon paste electrode (CPE), revealed that the nature of the bridging ligand and the overall structure of the complex highly influence the oxidation potential of Pd(II). At first, a complex of phenylpyridine with palladium (including insertion of Pd to C–H bond) and dialkyl phosphonate was formed, followed by anodic oxidation to give the final coupling product.

**Scheme 15:**
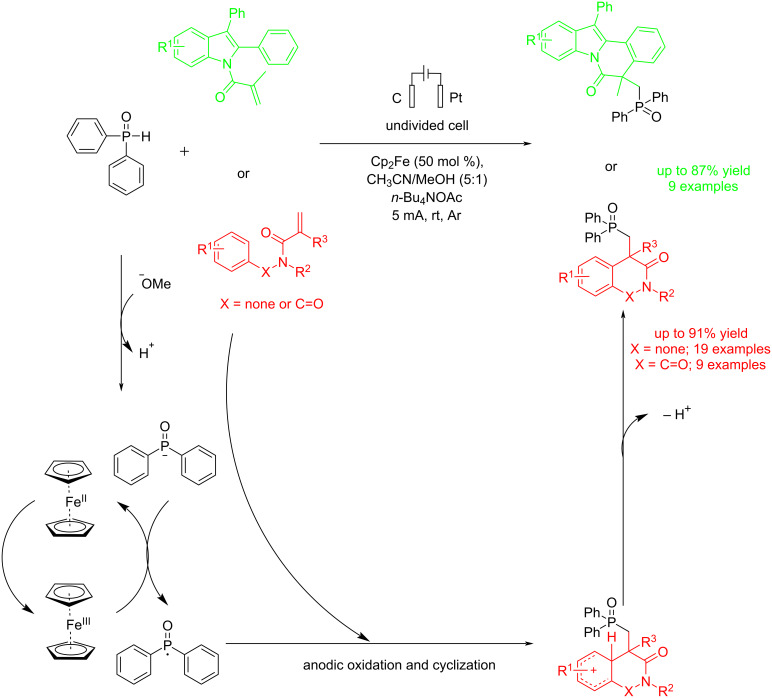
Electrochemical P–C bond formation of amides.

In 2023, Zhou et al. [59] reported an electrochemical method for the synthesis of phosphorylation of oxindoles and indolo[2,1-*a*]isoquinoline-6(5*H*)-ones using Cp_2_Fe through a radical addition/cyclization reaction at room temperature under argon gas. This research shows that this method is effective with various functional groups and can help to find new drug candidates. The reaction was carried out in an undivided cell where the anode was platinum, and the cathode was graphite at a constant current of 5 mA. The mechanistic study showed that a radical process might be involved in the reaction, and the role of phosphorus-centered radical intermediates was confirmed. The importance of Cp_2_Fe and the amount of diphenylphosphine oxide became evident when a significant decrease in reaction yield was observed both in the absence of Cp_2_Fe (Table 5) and when a lower amount of diphenylphosphine oxide was used. Both electron-donating and electron-withdrawing groups produced products with yields ranging from 71% to 91%, and they were found to be effective in forming the corresponding polycyclic products (Scheme 15). Through cyclic voltammetry experiments, the researchers confirmed that since the oxidation potential of Cp₂Fe is lower than that of other substances, it is most likely oxidized first.

**Table 5:** Optimization studies.

Variation from the standard conditions	Yield (%)

without Cp_2_Fe	<10
MnCl_2_ instead of Cp_2_Fe	23
CH_3_CN/HOAc instead of CH_3_CN/MeOH	11
without electricity	n.r.

**Scheme 16:**
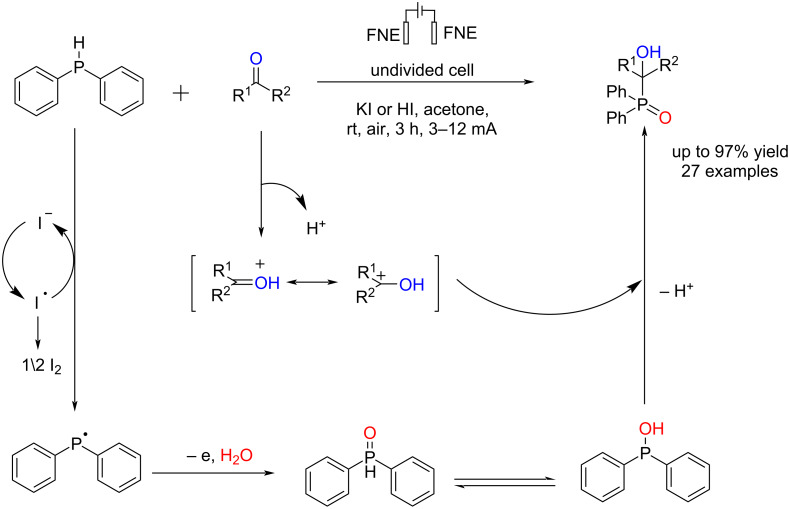
Electrochemical synthesis of α-hydroxy phosphine oxides.

In 2023, Ma et al. [60] reported an electrochemically oxidative/metal catalyst-free method for the synthesis of the α-hydroxyphosphine oxides through the reaction of diphenylphosphine as a phosphine source with aldehydes or ketones. They used nickel foam as both anode and cathode electrodes in an undivided cell under air at room temperature. The reaction was carried out in the presence of KI as an electrolyte, a key additive, and acetone as a solvent. HI increases the reaction yield due to the activation of the carbonyl group. The halide salts did not lead to product formation, indicating that chloride and bromide anions cannot generate the corresponding radicals to accelerate the conversion of diphenylphosphine. The reaction yield decreased when the methyl group was placed in *ortho*-position. Moreover, the desired products were obtained with moderate yields for aldehydes containing strong electron-withdrawing groups, indicating that this method is suitable for forming P–C bonds. Products with nitro, chlorine, or bromine groups at the *para* position had higher yields compared to those with the groups at the *meta* or *ortho* positions, which may be due to steric and electronic effects. The reaction proceeded via a radical process by forming Ph_2_P(O)H (Scheme 16). The reaction failed to give the corresponding product when using TEMPO in the reaction mixture.

**Scheme 17:**
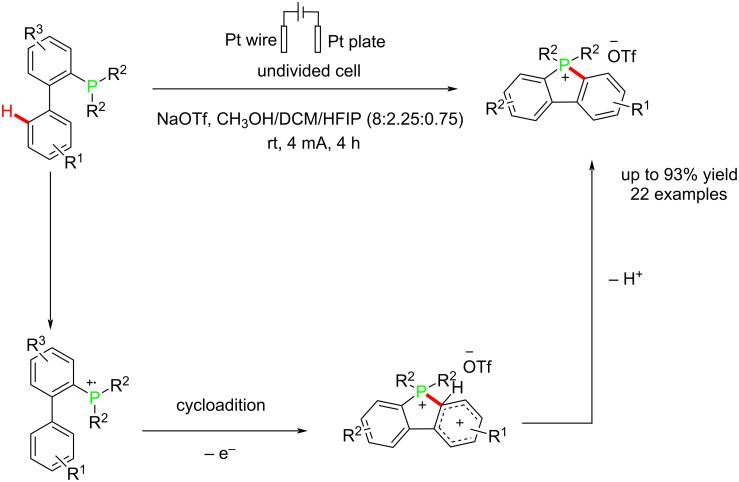
Electrochemical synthesis of π-conjugated phosphonium salts.

Zhang and co-workers [61] reported an electrochemical process for synthesizing π-conjugated phosphonium salts at room temperature in catalyst/oxidant-free conditions. The reaction was carried out in an undivided cell using a platinum plate as the anode and platinum wire as the cathode at a constant current of 4 mA. The reaction proceeded via an anodic oxidation followed by an internal combination of the formed radical to give the corresponding product (Scheme 17). The oxidation reaction was probably ineffective in the absence of HFIP due to the stability of HFIP's radical cation ions. The efficiency of the reaction was dependent on the electrolyte concentration, with a decrease in efficiency observed at lower concentrations. The reaction proceeded well under nitrogen, indicating that the oxidation process is unrelated to the presence or absence of oxygen. The yields of some products are likely due to the strong electron-withdrawing effects of the electron-withdrawing groups.

**Scheme 18:**
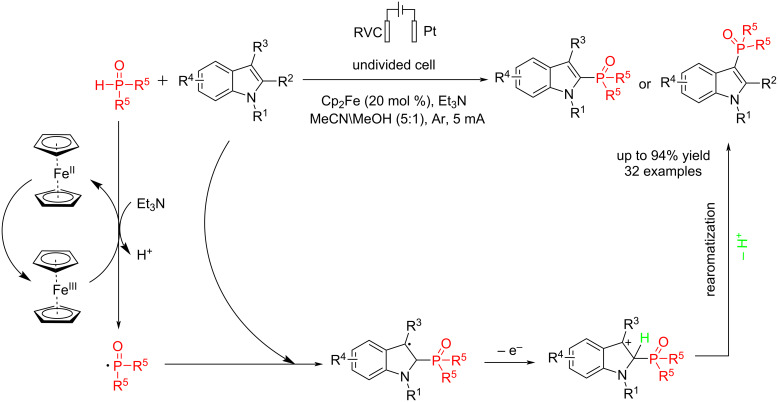
Electrochemical phosphorylation of indoles.

In 2024, Wang et al. [62] reported an electrochemical method for the synthesis of phosphorylated indoles in the presence of Cp_2_Fe as the mediator under mild reaction conditions without the need for external oxidants. This method improves the scalability of the resulting products, which also exhibit enhanced anticancer activity. The reaction was carried out in an undivided cell where the anode was reticulated vitreous carbon (RVC), and the cathode was platinum. Mechanistic studies revealed that Cp_2_Fe plays the main role in the reaction, and the reaction did not proceed without using Cp_2_Fe. Replacing the Boc group with an acetyl group significantly decreased the reaction yield. Furthermore, the results showed that the reaction proceeded via a radical phosphorylation process (Scheme 18). Cyclic voltammetry experiments demonstrated that Cp₂Fe is likely to undergo oxidation first due to its lowest oxidation potential among the species.

**Scheme 19:**
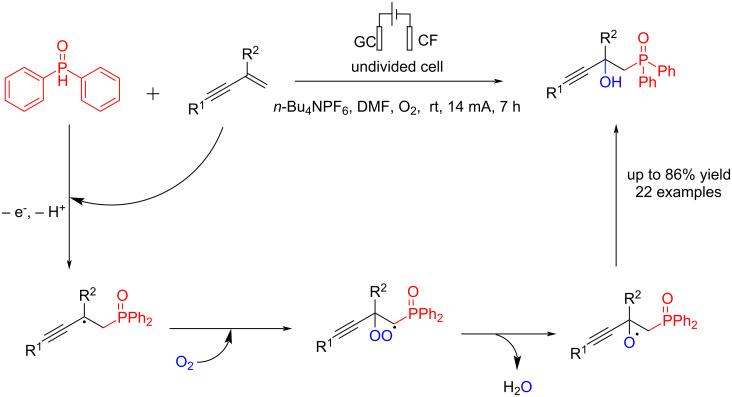
Electrochemical synthesis of phosphorylated propargyl alcohols.

In 2024, Zhu et al. [63] reported an electrochemical transition-metal and additive-free synthesis of phosphorylated propargyl alcohols at room temperature. The reaction is carried out in an undivided cell using glassy carbon (GC) as an anode and copper foam (CF) as a cathode at a constant current of 14 mA. Experiments confirmed that the trace amount of copper dissolved from the cathode had no catalytic effect on the reaction. The reaction proceeded via anodic oxidation of diphenylphosphine followed by a reaction with alkenes to give corresponding phosphorylated propargyl alcohols (Scheme 19). The reaction yield showed that this method is not sensitive to electron-withdrawing or electron-donating groups at different positions on the aromatic ring. Most likely, the 3-substituted pyridine substrate and the enynes with nitro or carbonyl groups on the aromatic ring did not react in this system due to the incompatibility of the intermediate radicals.

#### Electrochemical N–P bond formation

**Scheme 20:**
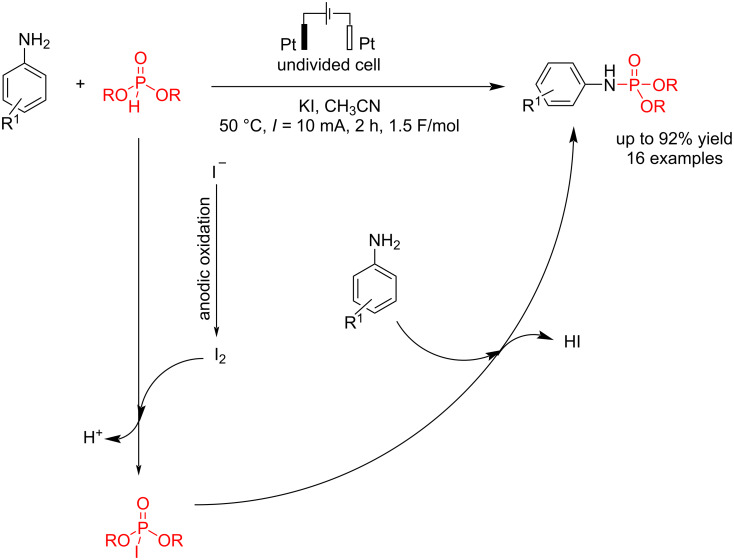
Electrochemical synthesis of phosphoramidates.

Due to the importance of phosphoramidates in medicine and organic synthesis, Zhong et al. [64] reported an electrochemical P–N coupling of amines with dialkyl phosphonates for synthesizing phosphoramidates. The reaction was carried out in an undivided cell at a constant current of 10 mA using platinum electrodes as the anode and cathode and potassium iodide as a key additive. Studies have shown that the choice of solvent significantly impacts the reaction. Studies have shown that the choice of solvent significantly impacts the reaction. CH₃CN exhibits better than CH₃OH, with a wider electrochemical window and better reactant solubility. When the reaction was conducted in CH₃CN using *n*-Bu₄NPF₆ and *n*-Bu₄NBF₄ as electrolytes but without iodide salt and in the presence of air, no product was formed. This finding indicates that iodide salt plays a crucial role in driving the reaction and acts as a catalyst in the reaction process**.** The electronic properties of the substituents on the compounds influenced the reaction yield. Phenol with the –OMe group produced a lower yield than the –Me group. This decrease in yield is likely due to the lower oxidation voltage of the –OMe group, which may lead to the formation of unwanted byproducts. The reaction began with an anodic oxidation of iodide to iodine, followed by a reaction with dialkyl phosphonate to give I–P(O)(OR)_2_. The final product was formed by a simple nucleophilic substitution of the phosphorus center (Scheme 20).

**Scheme 21:**
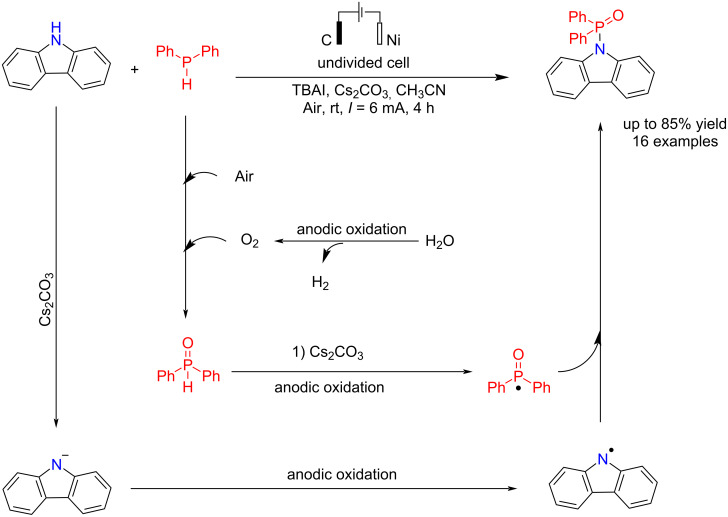
Electrochemical reaction of carbazole with diphenylphosphine.

The N–P bond formation is a critical process in organic synthesis due to the preparation of various materials with different biological and medicinal activities. In 2021 Wang et al*.* [65] reported a novel method for the N–P bond formation of carbazoles with diphenylphosphine using an electrochemical process. The main advantage of this method is its high selectivity, as only a 1:1 ratio of the starting materials was required for the reaction. The reaction was carried out in an undivided cell using TBAI as a key additive and carbon and nickel electrodes as the anode and cathode for 4 h at a constant current. Graphite, platinum, nickel, and reticulated vitreous carbon (RVC) electrodes were examined in this conversion. The platinum electrode did not perform well in the anode, and no reaction was performed. The use of graphite and RVC gave good results. Nickel, graphite, and platinum electrodes were examined as the cathodes. Results showed that platinum and nickel performed better than graphite in the cathode due to their higher conductivity and lower electrical resistance. When platinum and nickel were used as the cathode and graphite as the anode, the efficiencies of the processes were very close to each other. The use of dry acetonitrile as the reaction medium significantly reduced the yield. These results indicate that water plays a crucial role in this reaction, as its decomposition leads to the generation of O₂, the primary oxygen source in the reaction process. A radical process was proposed in the reaction. Diphenylphosphine oxide and carbazole radicals were formed via anodic oxidation in the presence of a base, followed by a coupling reaction to give the final P–N product (Scheme 21).

**Scheme 22:**
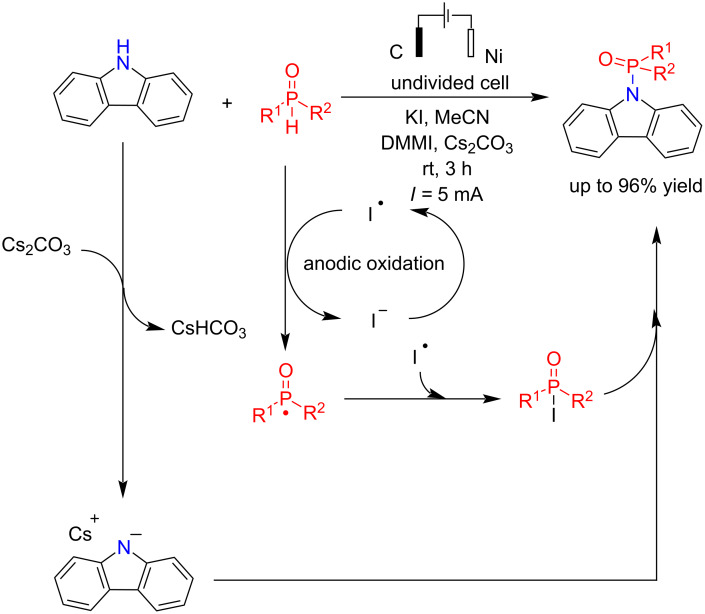
Electrochemical P–N coupling of carbazole with phosphine oxides.

In another attempt, Liu et al. [66] also reported the electrochemical phosphorylation of carbazoles and indoles in the presence of 1,3-dimethylimidazolium iodide (DMMI) as a mediator in the oxidation–reduction process. The reaction proceeded in an undivided cell using cesium carbonate as a base for 3 h with graphite and platinum electrodes as the anode and cathode, respectively (Scheme 22). In this reaction, a variety of substitutions were examined. The results showed that the reaction proceeded very well with electron-donating groups such as –OMe, –Me, and –CH_2_CN and electron-withdrawing groups such as –Cl, –Br, and –CO_2_Me. It was observed that carbazole derivatives with an extended conjugated system showed enhanced reactivity. Like the above P–N coupling mechanism, the reaction proceeded by an anodic oxidation of iodide to iodine followed by a reaction with dialkylphosphine oxide to give I–P(O)(R)_2_. The exact mechanism of this coupling reaction is not yet fully understood; however, the possibility of direct radical cross-coupling between the nitrogen radical derived from carbazole and the phosphoryl radical intermediate cannot be completely ruled out.

**Scheme 23:**
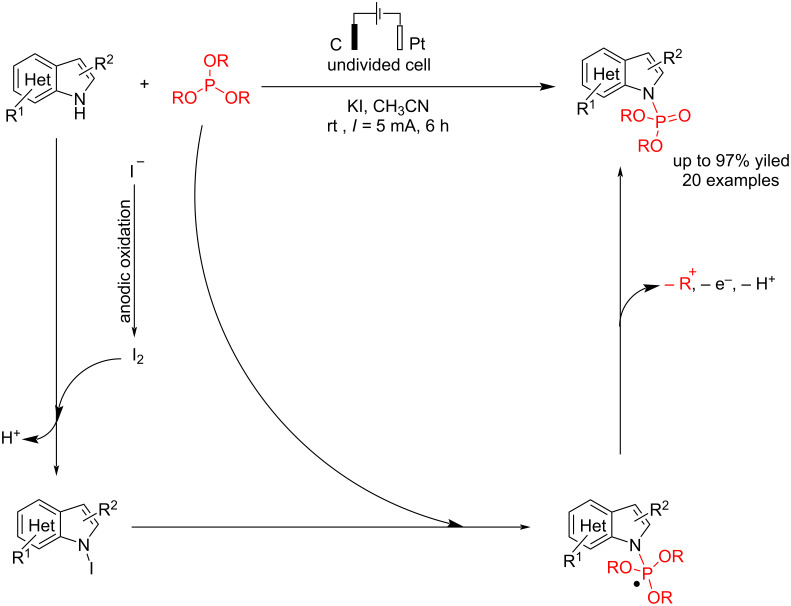
Electrochemical P–N coupling of indoles with a trialkyl phosphite.

In another electrosynthesis report, Gao et al. [55] reported similar P–N coupling reactions of indoles with trialkyl phosphites in the presence of potassium iodide as a mediator and electrolyte. The carbon and platinum electrodes were used as the anode and cathode at a constant current of 5 mA for 6 h in acetonitrile as solvent. The results showed that potassium iodide is critical in this reaction, and the reaction failed to give a corresponding product without using KI. This reaction was quickly extended to a wide range of substituted indoles. Moreover, despite significant steric hindrance or the presence of a long alkyl chain, both P(OiPr)_3_ and P(O*n-*Bu)_3_ proved effective in this reaction. In this reaction, the P–N coupling process proceeded via forming an *N*-indole iodide intermediate via anodic oxidation of iodide to iodine, followed by a reaction with indole (Scheme 23). Cyclic voltammetry demonstrated that in the presence of *n*-Bu₄NClO₄, free indole undergoes oxidation due to its electron-rich nature, forming a radical-cation intermediate. However, when KI was used instead, oxidation of free indole was not observed, indicating a different oxidation pathway. Additionally, under certain low-yield conditions, the C3-phosphorylated product was also formed.

**Scheme 24:**
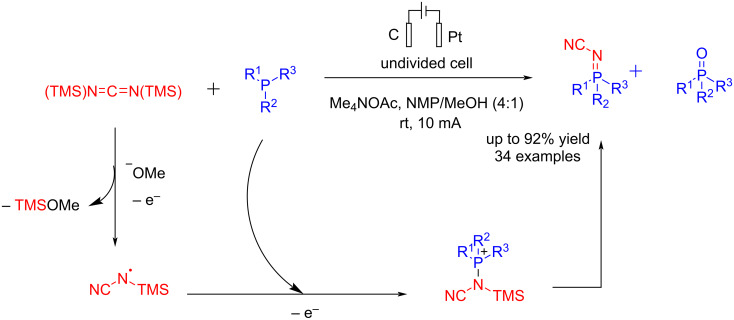
Electrochemical synthesis of iminophosphoranes.

In 2024, Mdluli et al. [67] reported an electrochemical method for synthesizing iminophosphoranes. In this method, iminophosphorane was investigated due to its air stability and the presence of a UV–vis chromophore, which enables the analysis of the reaction via UPLC. This reaction was carried out in an undivided cell using graphite as the anode and platinum foil as the cathode at a constant current of 10 mA. In the presence of a graphite anode, a stainless-steel cathode, and Et₄NBr as the electrolyte, the oxidation of PPh₃ was observed. For optimization of the reaction, an HT*e*^−^Chem reactor was used in the presence of Me₄NOAc as the electrolyte. The use of NMP and Me₄NOAc was beneficial, as in many cases, adding water led to the precipitation of the desired product from the reaction mixture. A wide range of iminophosphoranes were synthesized according to the following mechanism via an anodic trimethylsilyl cyanide radical formation (Scheme 24). The formation of the Ph₃P=O as the side product was assumed to be due to the presence of water or oxygen in the reaction mixture, which competes with the aminating reagent.

#### Electrochemical O–P bond formation

**Scheme 25:**
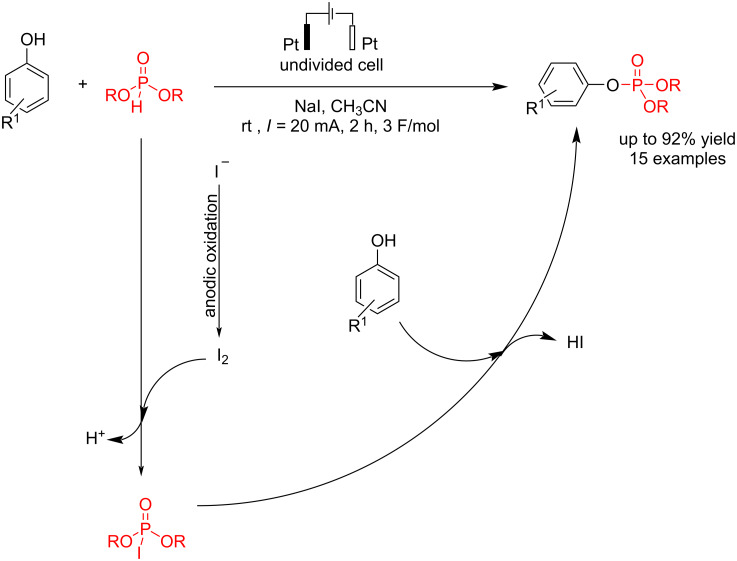
Electrochemical P–O coupling of phenols with dialkyl phosphonate.

In 2021, Zhong et al. [64] reported an electrochemical coupling reaction of phenols with dialkyl phosphonates. The reaction was carried out in an undivided cell using platinum electrodes in the presence of sodium iodide at a constant current. Various electrodes were examined, and the best results were obtained using platinum electrodes as the anode and cathode (Scheme 25). Studies showed that CH₃CN performed better than CH₃OH due to its wider electrochemical window and better solubility. The results indicated that the electronic properties of substituents had no significant effect on the yield, and all substituted anilines afforded high yields of phosphoramidates. However, *N*-methyl- and *N*-ethylanilines showed lower yields and electron-donating groups led to a reduced yield compared to unsubstituted aniline. Further, KI performed best as the electrolyte and catalyst at 50 °C (Scheme 20). The reaction proceeded with anodic oxidation of iodide to iodine, followed by a reaction with dialkyl phosphite to give I–P(O)(OR)_2_. The final product was formed by a simple nucleophilic substitution of phenols with I–P(O)(OR)_2_.

**Scheme 26:**
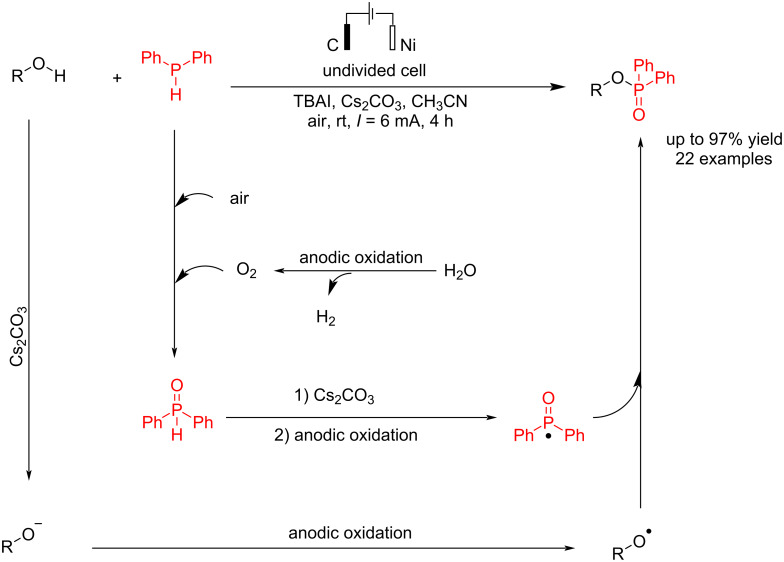
Electrochemical P–O coupling of alcohols with diphenylphosphine.

In 2021, Wang et al. [65] presented a report on electrochemical P–O bond formation. In this method, they have reported an electrochemical reaction of alcohols (aliphatic and aromatic) and diphenylphosphine in an undivided cell, using carbon and nickel electrodes as the anode and cathode, respectively, at a constant current for 4 h in the presence of cesium carbonate as a base. Various phenols, including those with electron-neutral, electron-donating, and electron-withdrawing groups, were efficiently converted into the target products in high yields. Phenols containing condensed aromatic and heterocyclic rings were also identified as suitable starting materials. The reaction proceeded via the anodic oxidation of diarylphosphines to diarylphosphine oxides, followed by further anodic oxidation to give phosphine oxide radicals. In the subsequent step treatment of alkoxide radicals (formed via the deprotonation of alcohols with cesium carbonate and anodic oxidation), the phosphine oxide radicals gave a corresponding phosphorylated products (Scheme 26).

#### Electrochemical S–P bond formation

**Scheme 27:**
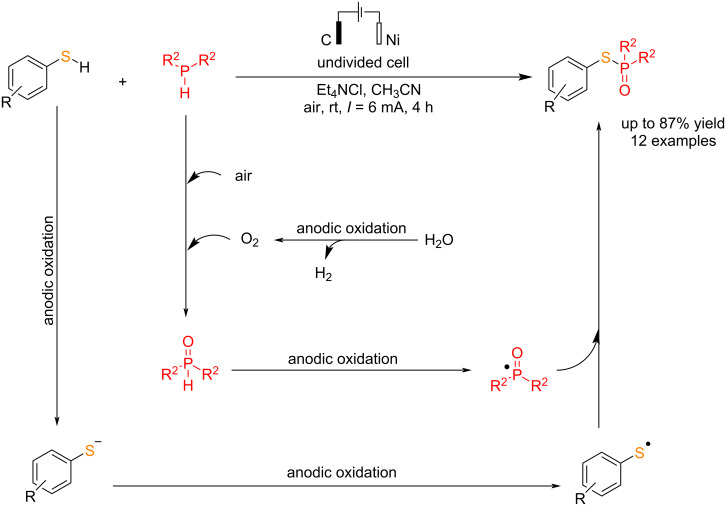
Electrochemical P–S coupling of thiols with dialkylphosphines.

Phosphorothioates are essential compounds with broad applications in industry and medicine. Wang et al. [65] reported a novel electrochemical process for direct P–S coupling of thiols with dialkylphosphines. The reaction was carried out in an undivided cell using carbon and nickel electrodes as the anode and cathode in a constant current for 4 h. They have prepared various phosphorothioates in moderate to good yields. Methanol, as an environmentally friendly solvent, provided a satisfactory outcome. Changing the electric current intensity reduced the yield, and using dry acetonitrile also led to a significant decrease in yield. These results indicate that water undergoes decomposition in the reaction, generating oxygen as the primary oxygen source in the system. The reaction proceeded via the anodic oxidation of phosphines to phosphine oxides, followed by further anodic oxidation to give a phosphine oxide radical. In the subsequent step, treatment of the thiol radical (formed via the deprotonation of thiols with cesium carbonate and anodic oxidation) with the phosphine oxide radical gave the corresponding thiophosphorylated product (Scheme 27).

**Scheme 28:**
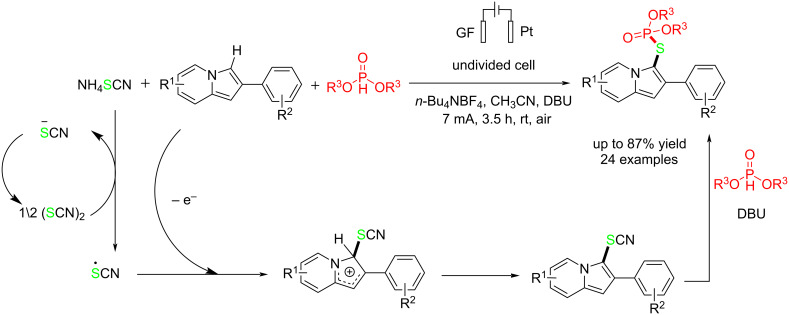
Electrochemical thiophosphorylation of indolizines.

Recently, a novel and one-pot electrochemical oxidation method was reported for synthesizing *S*-(hetero)aryl phosphorothioates without using any oxidants or transition metals at room temperature [68]. The reaction was carried out in an undivided cell. The anode and cathode electrodes were graphite felt (GF) and platinum, respectively, at a constant current of 7 mA. When a platinum plate or a carbon rod was used instead of GF for the anode, and a nickel plate or copper plate was used for the cathode, the efficiency of the reaction decreased (Table 6). Although using other solvents improved the solubility, it reduced the reaction yield. Ammonium thiocyanate is used as a sulfur source in the presence of DBU as a base and *n*-Bu_4_NBF_4_ as an electrolyte. When electron-donating groups were present at the C2-benzene ring, the reaction yield was higher than when the rings contained electron-withdrawing groups. It is suggested that the reaction proceeded via single-electron oxidation of thiocyanate at the anode. DBU was used in the reaction for a simple nucleophilic substitution of phosphonate with a cyanide group in the formed intermediate (Scheme 28).

**Table 6:** Optimization studies.

Variation from the standard conditions	Yield (%)

none	83
DMF as the solvent	36
without DBU	n.r.
Pt as the anode	78
C(rod) as the anode	69
Ni as the cathode	67
Cu as the cathode	56

**Scheme 29:**
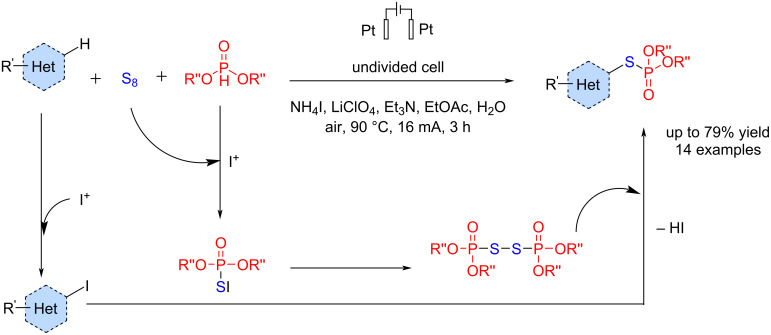
Electrosynthesis of *S*-heteroaryl phosphorothioates.

In another study, Ding and co-workers [69] recently reported an electrochemical method for synthesizing *S*-heteroaryl phosphorothioates without using any transition metal catalysts and oxidants at 90 °C. This method is compatible with various functional groups and can be easily scaled up to a gram scale. The reaction was carried out in an undivided cell using platinum electrodes as the anode and cathode. In this method, S_8_ was used as a sulfur source and ammonium iodide as a mediator, which has a key role. Based on the control experiments, a nucleophilic substitution is the main pathway for the reaction. The reaction proceeded with electrophilic iodination of aromatic compounds followed by a nucleophilic reaction of the formed phosphorothioate intermediate to give corresponding *S*-heteroaryl phosphorothioates in good to excellent yields (Scheme 29).

**Scheme 30:**
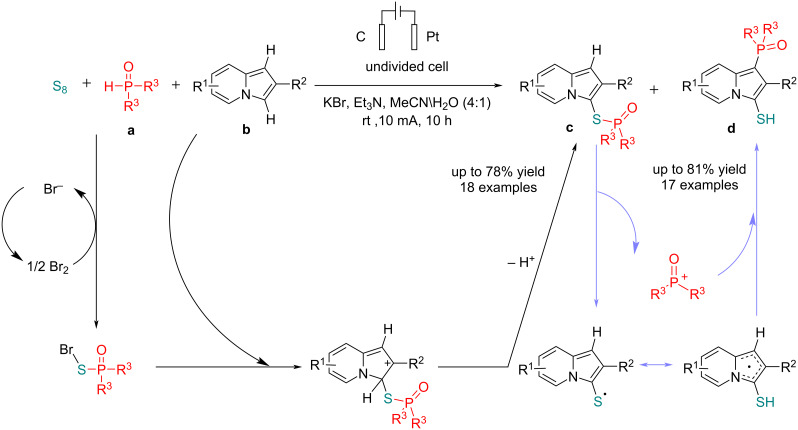
Electrochemical phosphorylation reactions.

In 2023, Liu et al. [70] reported an interesting electrochemical process for the simultaneous C–H phosphorothiolation and S- to C-[1,4]-phosphoryl migration at room temperature. The reaction was carried out in an undivided cell using a carbon plate as the anode and platinum as the cathode at a constant current of 10 mA in the presence of KBr as a key mediator. Using a mixture of solvents, product **c** was obtained with a lower yield, and product **d**, which was formed via the 1,4-S → C phospho-Fries migration of product **c**, was also obtained in low yield (Table 7). By reducing the current to 5 mA, a mixture of products was obtained, and it also caused issues in the reaction system. The reaction proceeded via an anodic oxidation of bromide to bromine, followed by a reaction with sulfur and dialkylphosphite to give the corresponding dialkylphsophothioate. The reaction proceeded via an electrophilic aromatic substitution in the next step to provide the final product (Scheme 30). The experimental results showed that product **c** was initially formed and then continuously transformed into product **d** via the phospho-Fries rearrangement. This transformation was completed in the presence of Et_3_N within 5 hours. Additionally, an excess of S_8_ and (EtO)_2_P(O)H likely inhibits the occurrence of this rearrangement.

**Table 7:** Optimization studies.

**a**/S_8_/**b**/base	Variation from the standard conditions	Yield **c** (%)	Yield **d** (%)

1:2.2:2.2:2.2	none	78	–
1:2.2:2.2:2.2	MeOH/H_2_O as the solvent	13	10
1:2.2:2.2:2.2	THF/H_2_O as the solvent	20	16
1:2.2:2.2:2.2	constant current = 5 mA	27	30
1:1.1:1.1:1.1	none	–	81
1:1.1:1.1:1.1	constant current = 5 mA	25	47

#### Electrochemical Se–P bond formation

**Scheme 31:**
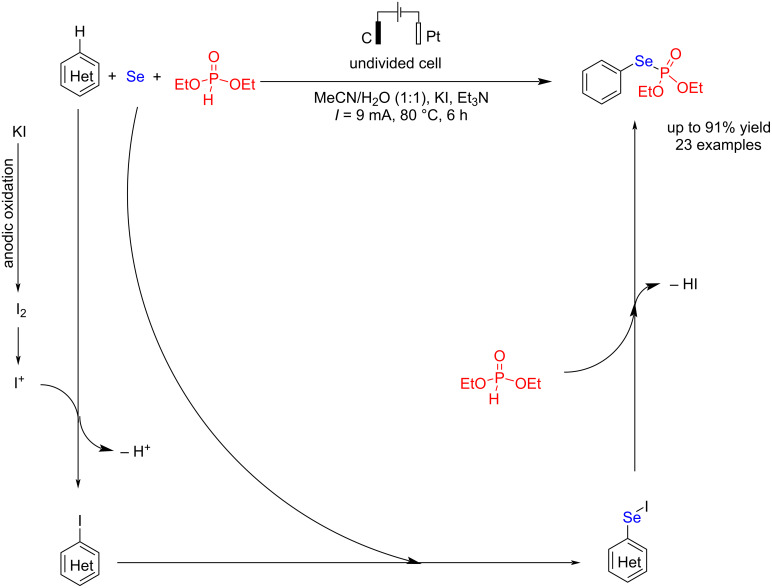
Electrochemical P–Se formation.

In another study, Gu et al. [71] reported electrochemical P–Se bond formation of the reaction of elemental Se with diethyl phosphonate and aromatic compounds. In this method, potassium iodide acts as a key additive. The reaction is carried out in an undivided cell using graphite and platinum electrodes. Using water as a co-solvent in the reaction was essential, as its absence led to a complete loss of efficiency. Lowering the reaction temperature and shortening the reaction time also reduced product yields. In this method, the target phosphoroselenoates were formed with moderate to high yields in the presence of electron-donating groups such as methyl (Me) and methoxy (OMe), as well as electron-withdrawing groups like fluorine (F), chlorine (Cl), bromine (Br), and nitro (NO_2_) at the 5-position of the phenyl ring in indole. Studies showed that even unprotected anilines participated in this reaction, yielding the desired product with a 72% yield. Furthermore, anilines bearing electron-donating groups such as methyl or weak electron-withdrawing groups like chlorine at the *ortho* position were efficiently involved in the electrochemical phosphoroselenylation reaction, producing the corresponding products with satisfactory yields. These findings demonstrate this method's broad substrate scope and high functional group compatibility. The P–Se bond formation process proceeded by forming an aryl iodide intermediate via anodic oxidation of iodide to iodine, followed by the iodination reaction of aromatic compounds (Scheme 31). In the next step, elemental selenium was inserted into the aryl iodide bond to form the aryl iodoselenide intermediate for forming the P–Se bond.

#### Other electrochemical reactions of organophosphorus

**Scheme 32:**
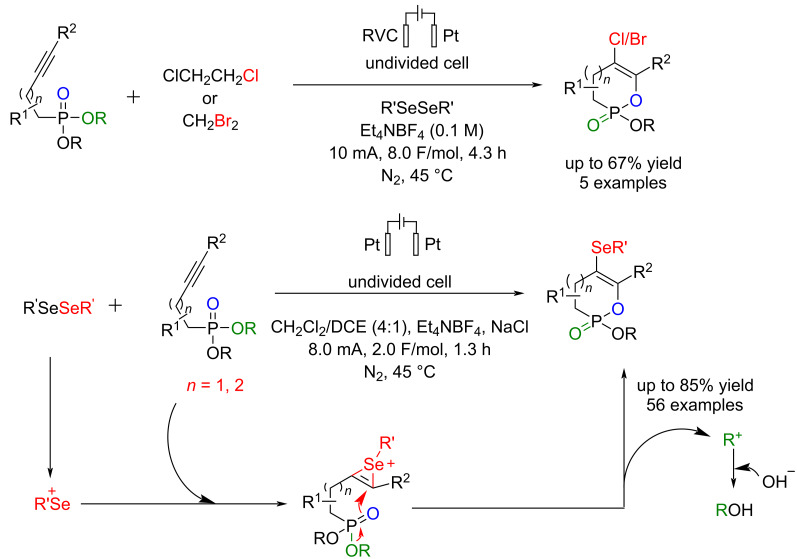
Electrochemical selenation/halogenation of alkynyl phosphonates.

**Scheme 33:**
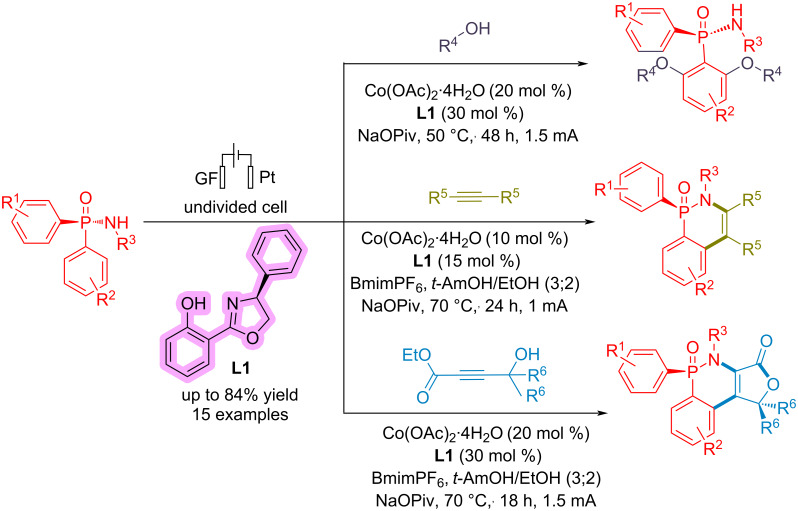
Electrochemical enantioselective aryl C–H bond activation.

Li et al. [72] reported an electrochemical synthesis method for the transition-metal-free cyclization and selenation/halogenation of alkynyl phosphonates at 45 °C under N_2_. This method can chemoselectively convert these products into halogen-functionalized cyclic enol phosphonates. The reaction is carried out in an undivided cell using platinum plates as the anode and cathode at a constant current of 8 mA. In addition to serving as an electrolyte, NaCl will likely facilitate the formation of PhSeCl, an active species in this reaction. The reactions proceeded smoothly, regardless of electron-donating or electron-withdrawing groups in the phenyl rings' *ortho*, *meta*, or *para* positions. This method proceeded via an anodic oxidation, followed by intramolecular cyclization (Scheme 32). The key role of anodic oxidation became evident when the annulation product was isolated exclusively from the anode chamber. Based on cyclic voltammetry experiments, the effect of diphenyl diselenide on the oxidative cyclization process was determined, showing that this compound enables the reaction to occur at a significantly lower electrode potential compared to what is required for the direct anodic oxidation of alkynyl phosphonates.

von Münchow and co-workers [73] reported an electrochemical process for enantioselective aryl C–H bond activation in the presence of a cobalt catalyst. The reaction is carried out in an undivided cell using graphite felt (GF) as an anode and platinum as the cathode at a constant current of 1–1.5 mA (Scheme 33). They did not present any mechanism for the reaction; however, based on their control experiments, they have suggested that electricity is necessary for the reaction, which makes the reductive elimination pathway easier, and Co undergoes oxidation state changes between Co(II), Co(III), and Co(IV).

## Conclusion

Organophosphorous compounds are important materials with a wide range of applications in industry, agrochemicals, and drug design. Therefore, introducing new methods for the preparation of these compounds will remain an interesting research area in organic reactions. This review focuses on electrochemical methods for forming various phosphorus–carbon, phosphorus–nitrogen, phosphorus–oxygen, phosphorus–sulfur, and phosphorus–selenium bonds. The main goal of the electrosynthesis method is to introduce novel and green processes for synthesizing a wide range of organophosphorus compounds. Different types of electrodes were used, and graphite and platinum were the most used. Most of the reactions proceeded via an anodic oxidation of materials, followed by the reaction with other compounds to give the products.

## Data Availability

Data sharing is not applicable as no new data was generated or analyzed in this study.
